# Research Progress of Grassland Ecosystem Structure and Stability and Inspiration for Improving Its Service Capacity in the Karst Desertification Control

**DOI:** 10.3390/plants12040770

**Published:** 2023-02-08

**Authors:** Shuyu He, Kangning Xiong, Shuzhen Song, Yongkuan Chi, Jinzhong Fang, Chen He

**Affiliations:** 1 School of Karst Science, Guizhou Normal University, Guiyang 550001, China; 2 State Engineering Technology Institute for Karst Desertification Control of China, 116 Baoshan North Road, Guiyang 550001, China

**Keywords:** grassland ecosystem, structure, stability, ecosystem services, karst desertification control

## Abstract

The structure and stability of grassland ecosystems have a significant impact on biodiversity, material cycling and productivity for ecosystem services. However, the issue of the structure and stability of grassland ecosystems has not been systematically reviewed. Based on the Web of Science (WOS) and China National Knowledge Infrastructure (CNKI) databases, we used the systematic-review method and screened 133 papers to describe and analyze the frontiers of research into the structure and stability of grassland ecosystems. The research results showed that: (1) The number of articles about the structure and stability of grassland ecosystems is gradually increasing, and the research themes are becoming increasingly diverse. (2) There is a high degree of consistency between the study area and the spatial distribution of grassland. (3) Based on the changes in ecosystem patterns and their interrelationships with ecosystem processes, we reviewed the research progress and landmark results on the structure, stability, structure–stability relationship and their influencing factors of grassland ecosystems; among them, the study of structure is the main research focus (51.12%), followed by the study of the influencing factors of structure and stability (37.57%). (4) Key scientific questions on structural optimization, stability enhancement and harmonizing the relationship between structure and stability are explored. (5) Based on the background of karst desertification control (KDC) and its geographical characteristics, three insights are proposed to optimize the spatial allocation, enhance the stability of grassland for rocky desertification control and coordinate the regulation mechanism of grassland structure and stability. This study provided some references for grassland managers and relevant policy makers to optimize the structure and enhance the stability of grassland ecosystems. It also provided important insights to enhance the service capacity of grassland ecosystems in KDC.

## 1. Introduction

Constituting almost 40% of the terrestrial biosphere, grasslands provide the habitat for a great number of diverse animals and plants and contribute to the livelihoods of more than 1 billion people worldwide [1]. Under the growing impact of global climate change and unreasonable human activities, grasslands are facing major problems that threaten the sustainable development of grassland ecosystems, such as a sharp decline in biodiversity, pasture degradation and reduced supply capacity [2,3,4]. Therefore, how to promote the healthy development of grassland ecosystems and enhance their service capacity has become an urgent issue [5,6,7]. Grassland ecosystem services are influenced by multiple factors such as their structure and stability. Furthermore, trade-offs and synergies between ecosystem services often depend on their structural and functional interactions [8]. A reasonable ecosystem structure can improve ecosystem productivity; promote material cycling, energy flow and information transfer; and increase the provision capacity of ecosystem service [9]. This plays a very important role in the healthy development of ecosystems and in human well-being. Rational allocation of grassland structure is one of the main measures to improve the stability and resilience of grassland ecosystems [10]. However, while the global concept of sustainability continues to spread, irrational structural configurations of grasslands (unreasonable cropping patterns and unscientific pasture management, such as planting density, species or grazing methods, etc.) continue to exist, which not only undermines the gains of ecological restoration and conservation, but also exacerbates the conflict between ecological conservation and economic development [11]. In particular, large-scale cultivation-based rangelands generally have a single planting structure, low biodiversity, high vulnerability and low stability, making it difficult to create a cascade of ecosystem service benefits [12]. As a result, grassland ecosystem structure and structural optimization are gradually becoming a research priority, with a focus on component structure, spatial and temporal structure, nutrient structure and their driving factors [13,14]. Optimizing the structure of grassland ecosystems is an important measure to improve and maintain the service capacity of grassland ecosystems [15,16], to balance grassland ecology and farmers’ livelihoods, and to resolve the contradictory issues of grassland ecology and sustainable economic development [17].

Structural changes drive changes in stability in grassland ecosystems [18], which, in turn, change their ecological functions and the provisioning capacity of an ecosystem [19]. At present, there are many studies about revealing the mechanisms of species diversity on grassland stability through controlled experiments in grassland [20,21,22,23]. The application of basic principles and methods of biochemistry to quantitatively study nutrient limitation and nutrient balance in forage, as well as the regulation mechanism of water–fertilizer coupling on forage quality, productivity and stability in grassland, which is the focus of the current research [24,25,26]. At the same time, studies on assessing the stability of grassland ecosystems are gradually emerging [27], such as those using remote sensing data; indicators characterizing ecosystem vitality, resilience and organization; and landscape pattern indices to evaluate ecosystem stability [28,29]. However, there is a lack of research regarding the mechanisms by which the complementarity and diversity of functional traits regulate the stability of grasslands, which results in a structure–process–function–service cascade [30]. What is more, there are different methods for quantifying ecosystem stability indicators, and the evaluation models are uneven; therefore, whether they are universally applicable remains to be verified [31,32]. Biodiversity and species diversity have an important influence on the productivity, stability and nutrient cycling of grasslands and their resistance and resilience to disturbance. Resilience, resistance and restoration are the main elements in determining whether an ecosystem is stable [33]. The study of the role of biodiversity, species diversity and functional traits on stability has been the focus of ecosystem research [34,35,36,37], and they are influenced by multiple factors on multiple scales [38]. It has been shown that extreme weather and irrational human activities are the main drivers of structural changes in grassland ecosystems [39], which indirectly alter ecosystem stability, increase the overall vulnerability of grassland ecosystems and reduce their capacity to provide ecosystem services [40]. Therefore, a deep understanding of the relationship between the structure and stability of grassland ecosystems is a major part of maintaining the stability of grassland ecosystems and enhancing their service capacity [41,42,43].

Due to their fragile attributes, ecologically fragile areas are prone to high ecosystem sensitivity and structural vulnerability under climate change and irrational human activities, which, in turn, reduces the stability of their ecosystems and changes their ability to supply services [44]. Therefore, the mutual interaction between ecosystem structure and stability should be deeply understood to enhance their resistance to human disturbances and environmental changes [45,46]. However, in ecologically fragile areas, irrational land use reduces the diversity of grassland species and productivity stability and affects the sustainable development of the region [47]. Especially in the environmentally fragile areas of karst, unreasonable human activities have led to the degradation of vegetation, increased soil erosion, gradual exposure of rocks and degradation of land productivity, with the surface showing a visual evolution similar to that of a desert landscape [48,49]. For this reason, the first task in the comprehensive control of rocky desertification is to restore and re-establish vegetation. In order to solve the ecological problem of karst desertification, the Chinese government has carried out a lot of work on the issue of karst desertification in southwest China since 1989, such as grain for green and closing the land for reforestation (grass), etc. The area of rocky desertification generally exhibits a trend of “continuous net reduction” [50], which provides a Chinese solution to global greening [51,52,53,54]. However, the KDC ecosystem still suffers from its simple structure, incomplete system function, high ecosystem sensitivity, lagging ecosystem service capacity and difficulty in maintaining the results of rocky-desertification management [55]. The rugged and fragmented surface in the karst areas, coupled with the constraints of rocky desertification, has affected the biodiversity of grassland ecosystems, resulting in a homogeneous structure and low productivity for the grassland [56]. Therefore, how to maintain ecological stability and optimize the structure of the system in the grassland ecosystems of KDC has become a key issue to consolidate the achievements of rocky-desertification management, ensure the smooth flow of service supply and demand, and enhance the well-being of local people [57,58]. Therefore, the structure and stability of grassland ecosystems and their interactions are not only a global concern [59,60,61], but also an important element that needs attention in KDC [62,63]. In the natural vegetation succession, grasses are the pioneer plants for vegetation restoration and ecological improvement [64]. Meanwhile, artificial grass breeding can enrich grassland species diversity, improve grassland ecosystem stability, provide high-quality forage, reduce the risk of surface erosion, improve soil nutrient composition and provide multiple ecosystem services for humans [51,65,66]. Optimizing the spatial configuration of systems is an important means of improving the stability and resilience of grassland ecosystems [67]. Therefore, optimizing the structure of grassland ecosystems for KDC and enhancing their stability are of great significance in enhancing the service capacity of grassland ecosystems and promoting the sustainable development of the regional ecological environment.

Thus far, research on the structure and stability of grassland ecosystems is increasing and breakthroughs have been achieved. The structure and stability of grassland is an essential element to maintain the sustainable and healthy operation of grassland in KDC, which is an important part of its ecosystem. However, there is a lack of relevant studies on the structure and stability of grassland ecosystems in KDC. In view of this, based on the perspective of grassland-ecosystem pattern change and its relationship with ecosystem processes, and the systematic review method, this study systematically reviewed the main research progress and landmark results on the structure and stability of global grassland ecosystems, and summarized the key scientific issues on structure optimization, stability enhancement and the interaction between structure and stability, aiming to provide some insights into grassland-ecosystem structure optimization and stability enhancement in KDC. In this way, it can enhance the supply of grassland ecosystem services and promote the sustainable development of the regional ecological environment and social economy in KDC.

## 2. Results and Discussion

### 2.1. Research Characteristics

**Figure 1:**
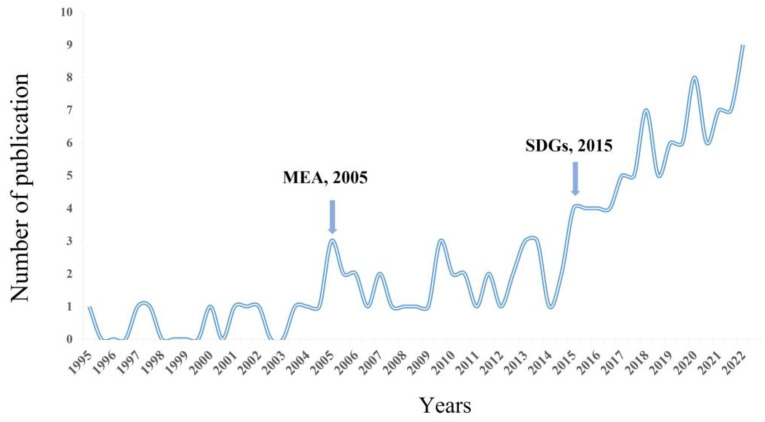
Trend in the annual distribution of literature related to the structure and stability of grassland ecosystems (MEA: Millennium Ecosystem Assessment; SDGs: Sustainable Development Goals).

As shown in Figure 1, the number of studies on the structure and stability of grassland ecosystems showed an overall fluctuating upward trend. From 1995 to 2004, the average annual number of articles did not exceed two, and it was in the budding stage. The number of papers published from 2005–2014 was 26, and the number of papers published during this period increased rapidly with the UN Millennium Ecosystem Assessment (MEA) synthesis report [68], with large fluctuations; this is the development phase. The number of articles published from 2015 to present was 97, mainly due to the convening of the United Nations Sustainable Development Goals (SDGs) [69]; the number of studies on ecosystem structure and stability shows a rapid upward trend, and the degree of research on concepts and mechanisms is gradually expanding and entering a diversified development stage.

#### 2.1.1. Stage Characteristics

##### Budding Stage

Research during the budding stage was mainly focused on theoretical studies of ecosystem structure and stability, and mechanisms of impact. The aim is mainly to improve the yield and quality of grassland forage through these studies and to meet the needs of human food security. The relationship between biodiversity and ecosystem function and productivity and the mechanisms that influence them have long been debated and have been shown to be positively correlated in national and international studies [70]. For example, through field experiments controlling for plant species diversity, functional diversity and functional composition, Tilman et al. concluded that these three factors are the main determinants of plant productivity, total plant nitrogen and light infiltration capacity, which, in turn, have a significant impact on ecosystems [71]. Spehn et al. monitored range biomass production, resource use (space, light and nitrogen) and decomposition processes on eight European pastures with different plant species richness, showing that higher species and functional group diversity also resulted in higher yields and more efficient use of resources [72]. These scholars have mainly observed changes in grassland biodiversity and productivity from the field scale, which is only a single-sided influence process, and have not addressed the interaction mechanisms between grassland species diversity, and biodiversity, etc., and stability. Moreover, the scale of the study is limited to the field scale, which does not involve the regional or global scale. Therefore, it remained uncertain at this stage how the findings would be extended to the landscape and regional level, and how they would be extended across different ecosystem types and processes [73].

##### Development Stage

Facing the threat of increased environmental pollution and the endangerment of cherished flora and fauna, the United Nations hosted the MEA, which motivated the conservation and sustainable development of ecosystems and was a phase of development in the study of the structure and stability of grassland ecosystems.

The study of factors that influence the structure and stability of grassland ecosystems was becoming progressively more advanced. How to manage pastures scientifically so that they can maintain healthy grassland ecology while balancing pasture production were the main issue of research during this period. For example, Isbell et al. conducted a long-term N enrichment experiment on grasslands to analyze the effects of N enrichment on productivity, plant diversity and the species composition of natural grasslands. The results showed that in the early stages, nitrogen enrichment increased grassland productivity, but over time, nitrogen enrichment was negatively correlated with species diversity and productivity [74]. Thus, scientific anthropogenic fertilization, generalizable results derived from experimental fertilization, is an important factor driving grassland biodiversity and species composition. Facing the problem that the productivity of natural grasslands cannot meet the current demand for livestock feed, the improvement of natural grasslands and the optimization of the planting structure of artificial grasslands became the focus of research at this stage [75]. For example, Albayrak et al. conducted experiments with seed mixes of oats and vetch on pastures in the highlands of Madagascar. The experiments showed that the mixed planting not only enhanced forage yield and quality, but also improved resource utilization [76]. Meanwhile, methods for evaluating the stability of grassland ecosystems are gradually arising. Zheng et al. used the fuzzy comprehensive evaluation method to evaluate the stability of mixed-seeded grassland in terms of community components, function and resistance to invasion. The results showed that the mixed seeding species and the proportion of mixed seeding could influence the community stability; however, this did not play a decisive role [77]. Therefore, how to scientifically quantify the stability of legume–grass mixed grassland communities, considering the temporal scale, spatial scale and their corresponding sensitive indicators, etc., is one of the issues that needed to be urgently explored at this stage.

In summary, research on the structure and stability of grassland ecosystems has gone through a budding stage and a development stage, which have developed towards diversification. In the budding stage, qualitative theoretical studies and influence mechanisms are the focus; in the development stage, with the aim of maintaining the stability of grassland ecosystems and protecting and promoting their sustainable development, a series of studies on the structure, function, stability influence mechanisms and stability evaluation of grassland ecosystems were carried out. Since then, the research directions and themes of ecosystem structure and stability have gradually developed in a diversified manner.

##### Diversification Stage

In order to thoroughly solve the development problems in the social, economic and environmental dimensions and shift to a sustainable development path, the United Nations Sustainable Development Summit put forward the SDGs, and many countries and regions have responded to the goals of poverty eradication, hunger eradication and climate change, etc. How to optimize the structure and stability of grassland ecosystems to promote sustainable development of ecosystems has become an issue that needs to be solved at the stage of diversified development [78,79].

With the gradual progress of research, the research at the stage of diversified development is mainly focused on the control experiments of grassland, to reveal the influence mechanism of species diversity and stability [80], and to quantitatively study the nutrients and productivity of pasture, and the regulatory mechanism of stability [24]. In addition, with the development of technology, data disclosure and the rise of multiple research methods (GIS technology, etc.), different spatial and temporal comparative studies have been widely performed.

The exploration of the mechanisms influencing species configuration and productivity and stability is a prerequisite for exploring the scientific management of grasslands and improving their productivity and stability. For example, Prieto et al. studied the effects of grassland productivity and sustainable supply capacity through species and genetic diversity. The results show that the complementary effects of taxonomy and genetic diversity can increase productivity under conditions of multi-grass species configurations, which, in turn, increases the productivity and resilience of grasslands in the face of environmental hazards [81]. Quantitative studies of nutrient limitation and balance and water–fertilizer coupling in forages are important processes to improve forage quality, productivity, and resistance to invasion [23]. Niu et al. assessed the effect of nitrogen enrichment on the stability of semi-arid grassland ecosystems in northern China and its potential mechanisms by simulating atmospheric nitrogen enrichment. The results showed that community stability was non-linearly related to nitrogen enrichment and that this relationship was positively correlated with species asynchrony, species richness and species diversity, as well as the stability of dominant species and the stability of grassland functional groups [82]. Therefore, it is important to re-evaluate the mechanisms by which multiple levels of nitrogen deposition affect the stability of natural ecosystems to gain a deeper understanding of the multiple nutrient inputs to grasslands and their stability response mechanisms.

In summary, the optimization and stabilization of grassland ecosystem structure can provide important basic research to elucidate the mechanisms of the complementarity and functional diversity of functional traits of forage grasses, as well as the synergy and trade-off between productive and ecological functions of grassland, thus promoting the healthy and sustainable development of grassland. We believe that, based on the idea of cascading benefits of grassland ecosystem pattern, process, function, and services, we should explore the mechanisms of multi-species configurations or natural grassland improvement and stability maintenance, and clarify how the species configuration of grassland (leguminous–grass, annual–perennial, and deep-rooted–shallow-rooted, etc.) affects the nutrient flow of grassland, which, in turn, affects community stability, and ultimately promotes the synergistic development of grassland-ecosystem productivity and stability.

#### 2.1.2. Stage Characteristics

Table 1 illustrates the global distribution of studies related to the structure and stability of grassland ecosystems. China and the USA have the highest number of published literature, with over 20 articles, which shows the concern for global issues such as the sustainable development of grasslands and food security in those countries [5,83]. Developed countries such as Europe and Oceania also published a relatively significant amount of literature. In addition, countries such as Brazil and South Africa account for a relatively small number of publications.

Due to regional differences in natural economic and social conditions, research on grassland ecosystems has developed unevenly and has prominent regional characteristics. In terms of the number of publications, Asia accounts for 47.8%, which is related to national policy support and the attention of research institutions [84]. In the Asian region, China is the main publication country, with most of the research focus on large-scale natural grasslands in the provinces of Tibet, Inner Mongolia and Xinjiang [85], where the restoration of these grasslands is of great importance to China in realizing the “two mountains theory” of “Lucid waters and lush mountains are invaluable assets.” [86]. The emergence of global issues (global warming, land degradation and resource crises) has led to sustainable and green development being a crucial issue in the 21st century, as exemplified by the United Nations SDGs. As a result, publications focused on the restoration of grassland ecosystems are gradually increasing, with particular attention being paid by countries in North America. In addition, with a large proportion of grasslands in North and South America, particularly in the Pampas, which has always been an export area for beef cattle, the sustainability of grassland ecosystems determines the economic lifeblood of the entire region. Similarly, although Europe does not have the same area of grassland as Asia or the Americas, food mainly comes from pastureland due to dietary habits, etc. As a result, the stable output of pastureland is vital to Europe’s survival [87]. Oceania is the region with the highest exports of wool and dairy products, and the quality of forage is related to the value of trade in export commodities and is equally important to the economy of the region as a whole.

The low number of publications in Africa is mainly related to the economic development of Africa, where most of the countries are still developing countries and the primary concern is to solve the problem of food and maintain national security and stability. Thus, although the area of grassland in Africa is large, and the sustainable development of savannah, in particular, is of great importance for maintaining the global climate, it is mostly studied by developed countries, such as the USA and Denmark [88,89,90]. South Africa is one of the few African countries with a significant volume of publications due to its better economic conditions.

### 2.2. Research Progress and Major Landmark Results

**Figure 2:**
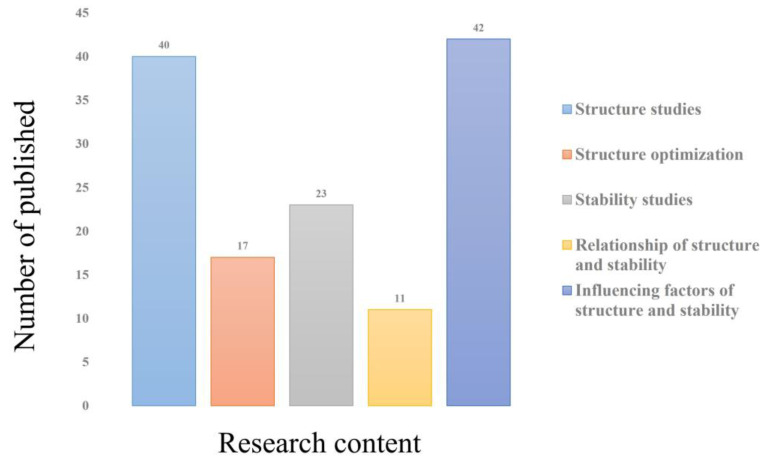
Grassland ecosystem structure and stability research content division. (From left to right, there is the legend of structure studies; structure optimization; stability studies; relationship between structure and stability; and influencing factors of structure and stability).

Based on the titles, keywords, abstracts and previous studies of the literature [91], drawing on the studies of Wang et al. and Fu et al. and the changes in grassland ecosystem patterns and their relationship with ecological processes [92,93], the 133 papers were divided into five aspects: structural studies (component structure, trophic structure, spatio-temporal structure), structural optimization, stability studies, structure–stability relationships and influencing factors (Figure 2), according to structural composition, ecological processes (replaced by stability) [94,95], the relationship between structure and stability, and factors influenced by the environment. The largest portion of literature focused on the factors influencing ecosystem structure and stability, at 31.5%, followed by structure studies, which accounted for 30% of this type of literature. In addition, studies on ecosystem stability accounted for 17.2% of the literature, while studies on ecosystem structure optimization and structure–stability relationships accounted for 12.7% and 8%, respectively.

Grasslands include the degraded, natural, and artificial, which determine the type of ecosystem structure and species composition of their habitats. This alters the process of ecosystem material cycling and information transfer through the component structure, spatial and temporal structure and nutrient structure [96], influencing the degree of ecosystem stability and, thus, the supply of ecosystem service capacity [97]. It also combines with artificial capital to form multiple forms of animal husbandry, which enter the market and, further, form an industrial chain which provides ecosystem services and benefits to humans. Therefore, attention to the structural composition and stability of grassland ecosystems is a prerequisite for interpreting the processes that shape grassland-ecosystem services.

#### 2.2.1. Structure Research

Grassland ecosystem structure refers to the relatively ordered and stable state of the various components of an ecosystem in space and time [98], including component structure, nutrient structure and spatial and temporal structure [99].

##### Component Structure

The structure of grassland ecosystems consists of biotic (plants, animals, micro-organisms) and abiotic (water, air, soil) factors, and a certain hierarchy and structural pattern is maintained between the components of each system. The composition of grasslands is the basis of all grassland research, and the analysis of changes in species composition and their response to grassland has always been an important part of grassland research [100,101,102]. Silva Mota et al. evaluated changes in floristic composition, structure, diversity, and life-forms spectra along an altitudinal gradient in the rupestrian grasslands in the south-eastern Espinhaço Mountains Range, and the results showed that differences in vegetation structure, diversity, species composition, frequency and the richness of each life form in relation to soil attributes and elevation. Plant height, species richness, diversity and evenness, frequency and richness of phanerophytes and chamaephytes decreased with elevation. The related results indicated that soil is an important driver of community change [103]. At the same time, human activities affect the species composition of grassland, and, thus, changing the cascading benefits of research into ecosystem services has become an emerging research direction. However, it is difficult to solve the various problems of grassland composition alone at the present stage. Therefore, the above studies were conducted in combination with spatial and temporal changes [104].

##### Spatial and Temporal Structure

The characteristics of ecosystems depend on biodiversity, that is, the functional characteristics of organisms present in the ecosystem, and the distribution and abundance of these organisms in space and time [105]. Due to species evolution and turnover, and a lack of scientific grassland management measures, the species diversity of grassland is gradually homogenizing, the soils are gradually being degraded, and the productivity is gradually decreasing [106]. Therefore, study on the spatial and temporal structure of grassland ecosystem provides a good reference for improving the service capacity of grassland ecosystem in the KDC area.

##### Time Structure

Quantitative assessment of the interannual variability in grassland landscape patterns in response to their ecosystem service values is a current research hot spot [107]. Monitoring the spatial and temporal dynamics of grassland areas can help decision makers to plan the use of grassland in a rational manner and achieve the sustainable development of grassland [108]. Landscape changes directly affect changes in the demand and supply of ecosystem services. Compared to the spatial scale of ecosystem services, little attention has been paid to how interannual changes in land use affects ecosystem service trade-offs, but this is necessary to facilitate the rationalization of decisions, especially when those decisions aim to re-establish diverse services and restore biodiversity [109]. Therefore, it is important to analyze the spatial and temporal evolution characteristics of grasslands and elucidate the main drivers of grassland-ecosystem service capacity to develop reasonable ecological restoration measures for grasslands. Recent studies have shown that current research has focused too much on global, national, and provincial spatial and temporal dynamics at large scales, neglecting research on the mechanisms of trade-off synergy and value transfer of grassland-ecosystem service functions at small scales in counties and sample sites [107]. Therefore, it is important to conduct overall monitoring of grassland landscape patterns in different periods in a specific region, analyze the temporal variability and heterogeneity of grassland-ecosystem landscapes at spatial scales, and grasp the structural-change characteristics of grassland ecosystems, to promote the optimization of the overall ecosystem structure and stability enhancement.

Seasonal changes in grasslands are also a current research hotspot in ecological restoration. The physical characteristics of plant growth in grassland determine its seasonal variation, which can lead to changes in ecosystem structure in the short term [110]. Thus, the changes in the species composition of grasslands in the short term can affect not only the above- and below-ground productivity supply of grasslands, but also the quality of forage and, in turn, the supply of multiple ecosystem functions. Rodriguez Barrera et al. compared the effects of groundhog and grassland use types and their seasonal changes on grassland vegetation structure and diversity in the semi-arid grassland in northern Mexico. The results of this study showed that grassland use type and its seasonal variation were the main determinants of grassland vegetation structure and cover [111]. However, current research on ecosystem services is mainly focused on inter-annual variability, with less research on monthly and seasonal variability [112]. Therefore, the interannual and seasonal changes in grassland-ecosystem structure should be strengthened in future studies.

##### Spatial Structure

Some studies have specifically classified grassland structure into vertical canopy structure and horizontal planting structure [113,114]. Therefore, the spatial structure of grassland ecosystems is discussed in terms of vertical and horizontal structure.

In terms of vertical structure, the three-dimensional structure of forest grassland is globally recognized as a stable state of ecosystems [115,116]. Studies have shown that a reasonable three-dimensional structure can effectively use light, heat, water, air and other environmental factors to improve productivity and maintain the stability of ecosystems [117], and that a composite ecosystem structure has a better soil retention effect [118]. The ecosystems of economic fruit forests and agroforestry are typical composite ecosystems, of which grassland is an essential and important component. The shallow roots of grassland combined with the deep roots of trees provide effective use of natural elements such as light and hot water and air, and intercept water through the multi-layered canopy, thus preventing flooding and providing resistance to erosion and, thus, having a good soil and water conservation effect [119].

In terms of horizontal structure, the optimal combination of multiple species can improve the productivity of grassland ecosystems and promote the efficient recovery of degraded grasslands, thus enhancing the stability and service functions of grassland ecosystems [120]. It has been proven that the persistence and stability of forage production in grassland ecosystems is generally higher than mixed planting with perennial grass species and single-forage cultivation [121,122]. Mixing planting with leguminous forages and replanting natural grasslands with leguminous forages can increase the productivity of grasslands, enhance their resilience and biodiversity, maintain the stability of grassland ecosystems and improve their service capacity [123]. By monitoring light cut-offs in the canopy of grassland communities with high species diversity, researchers found a positive correlation between plant species richness and multiple ecosystem functions, at which time grassland resource utilization was high. The relationship between grassland biodiversity-productivity and management intensity was also positively correlated when supplemented by sound grassland management [124]. Thus, a rational vertical canopy structure and a scientific horizontal structure not only maintain the biodiversity of the grassland, but also ensure its productivity, thus providing the basis for multi-functional grassland management [125]. In a protected area in central Italy (“Laghi di Suviana e Brasimone” regional park), Cervasio et al. showed that mixed seeding had a significant contribution to improving grassland quality and had a positive impact on grassland ecosystem biodiversity by changing the vertical and horizontal characteristics of grassland ecosystem species [126].

In addition, ecological corridors with a predominantly grassland landscape structure are also an important part of current spatial-structure research [127,128]. Ecological corridors are important links between the structure and function of a system, and grasslands can be used as buffer zones to improve the connectivity of ecological functions, allowing species to use corridors for long-distance dispersal or to provide shelter for some animal movements [129], thus maintaining and protecting biodiversity and improving the quality of their habitats [130,131]. At the same time, the spatial pattern of the landscape influences the magnitude of ecosystem service provision at the landscape scale through grassland use patterns and connectivity [132,133,134,135]. By quantifying inter-annual and seasonal changes in grassland landscape patterns in northern China, Hao et al. showed that degraded vegetation can influence changes in ecosystem service functions by changing land use patterns, increasing or dividing patch sizes of grassland and agricultural land, and increasing the size of forest areas where appropriate [136]. This is a very good reference for the grassland ecological protection of KDC. At this stage, however, most studies are still at the stage of qualitative description, and these mainly explore the factors influencing ecological functions (such as biomass production and other ecosystem services) and plant community characteristics [137]. There is also an urgent need to use these insights to develop combinations of grass species for high-yielding, high-quality communities [138], as the research subjects and geographical environments change and whether regional research results can be adapted to global environments or specific geographical areas, such as karst desertification areas and alpine grassland regions.

The study of the spatial structure of grassland ecosystems should focus on the vertical and horizontal cropping structure of grasslands and the distribution of grassland landscape patterns. This not only helps researchers to monitor the dynamics of grasslands comprehensively from a micro to macro level, but also helps grasslands (as an ecological linkage corridor) to provide better connectivity to other ecosystems, guaranteeing the healthy development of the whole terrestrial ecosystem and providing more and better service functions for humans [139]. Studies have found that increasing the abundance, evenness and diversity of dominant species not only effectively improves the productivity of the system [140,141], but also ensures the harmonious development of the “production-living-ecological function” of grassland ecosystems [142,143], maintaining the stability of their productive functions and the diversity of biological species. In summary, the vertical and horizontal structural characteristics of grasslands change the species composition and biodiversity of grasslands, affecting the quality of grassland habitats and their ability to provide services.

##### Trophic Structure

The study of trophic structure, food webs and ecological networks in ecosystems is the theoretical basis for understanding the composition, structure and dynamics of ecosystems, and provides indispensable scientific support for biodiversity conservation, ecosystem management and restoration, as well as response to global change [99]. The trophic structure of an ecosystem refers to the food chains and food webs formed by producers, consumers and decomposers in a biome with food as the link, which constitutes the main pathway for material cycling and energy flow [144]. Food webs are based on the interactions between species at different trophic levels, forming upstream and downstream regulatory mechanisms to maintain their structural stability [145].

Species interactions or alterations are one of the most important areas in the study of food webs. Food webs, which depict networks of trophic relationships in ecosystems, provide complex yet tractable depictions of biodiversity, species interactions, and ecosystem structure and function [146]. Nearly all ecosystems have been altered by human activities; these changes may modify species interactions and food-web stability [147]. De Castro et al. started from the classic soil food-web model of Hunt to formulate a plausible topology of soil food-web models and then compare the effect of this topology with those of random topologies [148,149]. The results showed that the stronger the species interactions in the soil, the more stable the food web, and that disrupting the functional-group assemblages and strength of interactions in the soil food web inevitably has a significant impact on species and their relative abundance. Duchardt et al. applied structural equation models (SEM) to disentangle direct and indirect effects of prairie dogs on multiple trophic levels (vegetation, arthropods, and birds) in the Thunder Basin National Grassland, and the results indicated that prairie dogs directly or indirectly influence associated vegetation, arthropods, and avifauna [150].

In summary, changes in key traits that sustain interactions are one of the most important factors in determining the stability of food webs [151]. Therefore, the study of species interactions is important for clarifying inter-species relationships and food-web driving mechanisms, which is one of the priorities that should be focused on in the future.

#### 2.2.2. Structure Optimization

Structural-optimization measures can change the species composition of grasslands, thus affecting their ecosystem biodiversity and altering their capacity to supply ecosystem functions. There are many ways to optimize structure, but this study will only discuss biological measures (optimal allocation of species structure) and engineering measures (pasture management systems).

**Figure 3:**
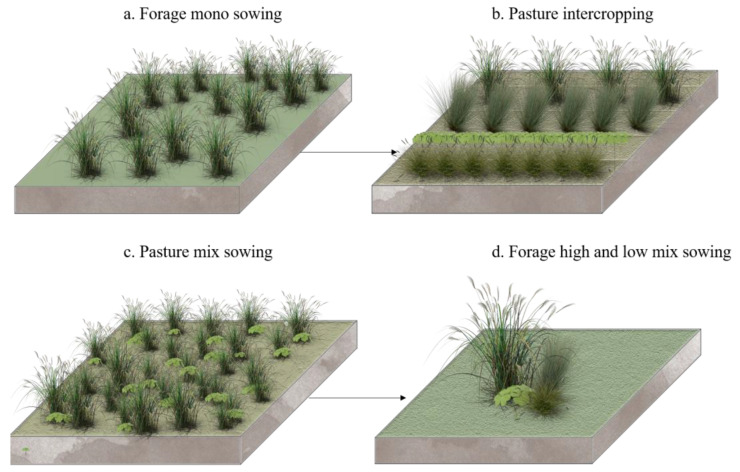
Artificial grassland planting structure. (**a**) Forage mono sowing: Grass forage, *Lolium perenne* L., (**b**) Pasture intercropping: Grasses-Leguminosae Forage, *Lolium perenne* L. + *Medicago sativa* L., *Trifolium repens* L.+ *Lolium perenne* L., (**c**) Pasture mix sowing: Grasses-Leguminosae Forage, *Lolium perenne* L. *+ Medicago sativa* L., (**d**) Forage high and low mix sowing: *Lolium perenne* L. + *Medicago sativa* L. + *Trifolium repens* L. + *Lolium perenne* L. (The grass species of (**b**–**d**) are the same, but the arrangement of planting order is different. (**b**,**c**) are planted in a horizontal way, but the density and proportion of planting are different. (**b**) is good for personnel to manage, and (**c**) is good for the nutrient complementation of grass species. (**d**) is planted in a vertical way, which can fully utilize light, heat, water and air).

Scientific and rational species allocation is the key to the high productivity and stability of grassland ecosystems [152]. Optimal species structure allocation includes the optimal vertical and horizontal allocation of grassland species. The three-dimensional structure of composite ecosystems has proven to be an important strategy for reconciling environmental protection and economic development in ecologically fragile areas [153], with grasslands being the most crucial aspect, and their strong renewal rate making them a large surface area within the composite ecosystem. The resulting three-dimensional ecosystem structure not only plays an important role in maintaining and providing ecosystem services, but its multiple ecosystem services also provide an effective way to promote the restoration of degraded ecosystems, which is an important biological measure to make full use of resources [117,154]. A diverse three-dimensional and horizontal structure is an effective measure to restore grassland ecosystems, that is, through a combination of different species of grasses from different families (in intercropping, crop rotation, and mixed sowing, etc.; see Figure 3) [155]. The most studied of these is the mixed cropping pattern of legumes and grasses. This mixed cropping pattern makes full use of sunlight, heat, water and air, creates complementarity between species, and promotes the uptake of soil nutrients by forage grasses, which, in turn, improves their annual growth and nitrogen use efficiency [156]. Multi-species planting with different habits is an effective means of restoring grassland ecosystems, and it can effectively improve the degradation of grasslands, protect and improve soil quality, enrich inter-species and intra-species species diversity, and effectively increase the resilience and recovery of degraded grassland ecosystems [157]. Scientific seeding is an effective measure to restore the dynamic balance of grass species [158]. Under the same circumstances, mixed-seeded grasslands have higher forage density and species richness, and it has more competitive and better grassland vegetation restoration compared to single-seeded grasslands. However, it has been demonstrated that species diversity and stability in grassland ecosystems with different rates of mixed seeding are positively correlated, and that seeding rates are negatively correlated with resistance to invasion [159]. Therefore, understanding forage seeding rates and their effective mix relationships is an important area of vegetation-restoration research [160]. Meanwhile, the mixing and intercropping of annual and perennial forages are also efficient management practices that take advantage of the temporal structure of grassland ecosystems, reducing inter-specific competition and increasing the persistence of forage production [161]. Mixing annual and perennial legume-grass is recognized as a highly productive and robust management practice [162], which can maintain a diverse supply of ecosystem services, and the leguminous forage grasses provide a more adequate supply of nitrogen to other grass species, thus maintaining high grass biomass and meeting the production requirements of farmers [163].

A scientific system of pasture management can effectively enhance the sustainable supply of grassland resources. Pasture management not only includes grass seed cultivation and fertilization, but also includes the full use of time and space, such as rotational grazing, rest grazing and ban grazing. In the early days, pasture management was mainly aimed at improving pasture production. In the new context of global climate change and increased environmental pollution, balancing ecological protection and green economic development has become a necessary path to improve the overall ecological quality of regions [164]. The general system of rotational and rest grazing is only an artificial strategic rest and tactical grazing of grasslands [165]. Therefore, some studies have proposed models such as the sustainable grazing system (SGS), DairyMod, GRAZPLAN, GrassGro, DairyNZ whole-farm model and EverGraze [166,167,168,169,170,171,172]. The above systems encourage the sustainable development of grassland ecosystems, and are key to preventing soil erosion and promoting soil improvement, as well as being important measures to conserve grassland plant biodiversity. Michalk et al. detailed sustainable and permanent grazing systems and answer the key questions currently facing Australia: (1) whether increasing the number of paddocks and implementing rotational grazing results in higher grazing rates, higher yields per hectare and better economic benefits; (2) which combination of grazing methods and grazing rates is most appropriate to create higher and more stable perennial grasslands to improve yields and environmental benefits in different parts of the landscape; and (3) can landscape variability be identified, mapped and effectively managed on native grasslands in high-rainfall areas [173] ? These can provide some inspiration for grassland grazing and farmers’ livelihoods in KDC.

Therefore, optimizing the structural configuration of grasslands in terms of both biological and engineering measures is currently an unavoidable and important issue for improving the sustainable development of grasslands and economic development. It will not only solve the shortage of supply capacity for ecosystem services and promote regional economic development, but also issues related to the global supply of food.

#### 2.2.3. Stability Studies

Stability refers primarily to the ability of a community or ecosystem to maintain its original structural and functional state and resist disturbance after a disturbance, known as resistance stability, and the ability of a community or ecosystem to return to its original state after a disturbance, known as resilience stability.

Exploring the causes that affect the stability of grassland ecosystems and suggesting targeted restoration strategies for degraded outcomes is a major focus of current research [83]. Community stability increases species diversity, species heterogeneity and population size, so maintaining the long-term stability of communities is crucial to maintaining ecosystem function. Researchers have explored the potential drivers and mechanisms of ecosystem stability by studying changes in the relationship between biodiversity and ecosystem service functions [174,175,176]. Biodiversity is a major driving factor of ecosystem stability [177]. Biodiversity consists mainly of above- and below-ground components. In terms of above-ground biodiversity, an increasing number of studies have shown that the more above-ground biodiversity (plant diversity) there is, the more stable the ecosystem is [178]. The results of Garcia-Palacios et al. showed that the diversity of leaf traits may promote the stability of an ecosystem under low drought conditions, while the species richness may play a greater role in the stability of an ecosystem under high drought conditions [179]. Similarly, below-ground biodiversity (soil biodiversity) altered grassland-ecosystem resistance and resilience through direct and indirect effects on plant diversity, net ecosystem productivity and plant species interactions, and, thus, changed grassland ecosystem stability [180]. In addition, recent studies have shown that phenological variation can also reconcile plant diversity with the seasonal stability of ecosystems, and, thus, affect the stability of the whole ecosystem [181].

Evaluating grassland-ecosystem stability has also become an effective means of detecting changes in grassland landscapes or the effectiveness of grassland restoration [182]. When evaluating the stability of grassland ecosystems, timescale variation is a factor that must be considered. By comparing the changes (years, seasons, or months) in grassland landscape patterns in different periods combined with evaluation models and methods, researchers have selected indicators of ecosystem vitality, resistance, resilience and variability to evaluate ecosystem stability [32], revealing the processes of change in grassland ecosystems under extreme weather conditions and their driver factors, quantifying the correlation between grassland ecosystem stability and biodiversity and their spatial patterns, and providing important prerequisites for improving sustainable ecosystem services [183,184]. The traditional evaluation methods of ecosystem stability are mainly qualitative analysis methods, including empirical methods and expert consultation methods, which are, generally, using expert consultation methods to quantify measures. The other is mainly quantitative evaluation including the comprehensive-evaluation method, gray-information analysis, ecological models and other methods, among which the most common methods of comprehensive analysis are hierarchical analysis, the entropy weighting method, the weighted comprehensive average method, and the comprehensive index evaluation method, etc. New kinds of ecosystem stability evaluation are diverse, and an increasing number of methods are becoming more credible and scientific in their assessment of ecological stability. These methods for evaluating the stability of ecosystems can provide good theoretical references for the ecosystem evaluation of KDC, and they can provide insights into the establishment of stability evaluation index systems for karst grassland ecosystems.

#### 2.2.4. The Relationship between Structure and Stability

A good system structure maintains its relative stability, and ecosystems with high stability generally have a more rational structure. Tilman et al. argued that diversity leads to higher community productivity, ecosystem stability and resistance to invasion [74]. Before the 1970s, ecologists believed that communities with higher diversity had more stable ecosystems [185]. Since then, ecologists have focused more on the relationship between species diversity and ecosystem stability [186,187].

Biodiversity affects ecosystem services by altering ecosystem function and stability [188]. Biodiversity–stability relationships showed that above-ground productivity and temporal stability increased significantly with increasing species richness, while biodiversity was largely influenced by ecosystem structure [85,189]. Therefore, understanding the relationship between grassland-ecosystem structure and stability and its influencing factors is essential for the sustainable development of grassland ecosystems [190]. However, how plant species diversity regulates the stability of ecosystems (such as biomass reproduction and nutrient cycling) has become one of the challenging questions in ecology [191,192]. Experiments have demonstrated that the higher the complexity of the ecological network of grasslands, the higher the ecological stability, by influencing plant physiological conditions, as well as species generation, diversity and variation [193,194]. Of course, grassland-ecosystem stability is also vulnerable to the influence of ecosystem components (ecosystem species diversity, composition), climate change and anthropogenic activities, mainly in the form of lowering the productivity of grassland, weakening biodiversity and declining service functions, which, in turn, can affect grassland-ecosystem services [195]. Therefore, studying the structure of grassland ecosystems and their material and energy flows and cycles among different components, and exploring the relationship between grassland-ecosystem structure, function, and stability is one of the research areas that should be focused on in the future [196]. Recent studies have shown that the positive relationship between biodiversity and stability is also influenced by spatial scale [197]. Therefore, the study of the relationship between structure and stability should also consider different spatial scales and timescales.

#### 2.2.5. Factors Affecting Structure and Stability

**Figure 4:**
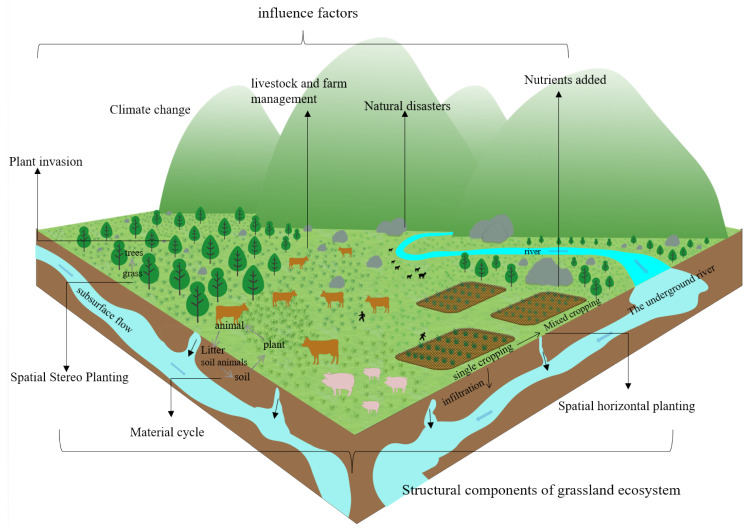
The structural components of grassland ecosystems and the factors that influence them combine to influence grassland ecosystem stability.

The structure and stability of grassland ecosystems are influenced by multiple factors, such as the natural environment and human activities. Therefore, the heterogeneity of spatial environments inevitably leads to variability in the degree of stability. In the case of grassland ecosystems, their structure and stability are mainly influenced by climate change, nitrogen deposition or nutrient addition, grazing and grassland management, plant invasion and natural disasters (Figure 4).

Global climate change is affecting ecosystem function and stability, indirectly altering species diversity, species composition, and functional plant traits [198,199], thus reducing the capacity of ecosystem services and reducing the various benefits that humans derive from them [200]. The impact of climate change on ecosystem function and biodiversity is highly dependent on grazing history and natural conditions [190]. By comparing changes in the spatial pattern of grasslands and simulating different climatic conditions on grassland ecosystem resistance, recovery times and rates, White et al. concluded that future climate change will have a significant impact on the resistance and recovery of disturbed ecosystems; however, the spatial pattern of impacts varies widely [201].

Key limiting elements can alter species interactions [202], change the spatial and temporal patterns of terrestrial plant communities [203], and influence ecosystem function [204]. Atmospheric nitrogen deposition and the application of nutrients (phosphorus, potassium, etc.) can affect species diversity in grassland communities [205]. The results of related studies show that the addition of nitrogen or phosphorus individually can increase the productivity of grasslands by 0–20%, while the addition of both nitrogen and phosphorus can increase the productivity of grasslands by 60% [206,207]. Nitrogen is an essential nutrient for plant growth, and an appropriate addition of nitrogen deposition will directly promote rapid plant growth and increase the net primary productivity of terrestrial ecosystems. However, excessive nitrogen deposition can also lead to soil acidification, altering the effectiveness of soil mineral nutrients and the structure and function of microbial communities, which, in turn, affects plant growth and changes plant diversity [208,209,210]. Plant diversity is closely related to ecosystem stability and productivity [211], and ultimately drives changes in ecosystem structure and function [212]. Therefore, paying attention to the differences in supply between different nutrient elements and the resulting differences in the resistance and resilience of plant diversity that result is extremely important for both productivity stability and the temporal stability of grasslands [213].

Plant invasion and vegetation succession are also among the factors that alter the species composition of grasslands and directly change the structure of grassland ecosystems [214,215]. Since the early 20th century, the invasion of woody plants into grasslands and their impact on the carrying capacity of grasslands has become a serious problem for savannas [216]. As reported in savanna ecosystems, the large-scale invasion of shrubs, trees, and other plants has led to significant changes in the functioning of global ecosystems, which will have profound impacts on the biodiversity, carbon storage capacity, and supply of these ecosystems [217]. Invasions of poisonous weeds has been considered one of the most important causes of economic losses by inhibiting forage production and killing livestock. However, a recent study concluded that toxic weeds can also have some potential positive ecological impacts on grasslands, such as promoting soil and water conservation, improving nutrient cycling and biodiversity, and protecting rangelands from excessive livestock damage [218]. Therefore, appropriate actions are needed by policy makers, managers and stakeholders to assess the ecological functions of invasive toxic weeds and to reconcile the long-term trade offs between livestock development and maintaining the ecological services provided by grasslands.

Animal husbandry plays an important role in eradicating hunger and improving malnutrition [219]. Protein and energy from livestock products are the main sources of human nutrition. Therefore, improving and sustaining livestock development is critical to advancing the United Nations SDGs, especially in addressing zero hunger and mitigating climate change [220]. Rational livestock management is a major initiative to promote healthy ecosystem development. Therefore, rangeland management, as a determinant of maintaining biodiversity, ecosystem services and landscape [221], is more important to focus on exploring its driving mechanisms on grassland species structure and ecosystem stability [222]. In addition, natural hazards are of great importance to our understanding of global biogenic burning and its emissions, carbon cycling, biodiversity, conservation and land management, especially as ecosystem succession and biodiversity changes associated with grassland fires are critical to the patterns and dynamics of ecosystem functions and services [223].

In summary, sustainable management of grasslands requires a deep understanding of the functional relationships among these factors due to the spatial heterogeneity of environmental conditions, production potential and flora composition [224]. It is more important to jointly explore the similarities and differences in the structural composition of grassland ecosystems in multiple factors and scales, extract the key factors affecting the service capacity of grassland ecosystems, and apply them to the restoration of the grassland vegetation of KDC.

## 3. Materials and Methods

Based on the research of Khan et al. and Chapman et al. [225,226], we performed a systematic literature search and review following the protocol from Preferred Reporting Items for Systematic Reviews and Meta-analyses (PRISMA) including quantitative statistics and qualitative content analysis. Systematic reviews have an advantage over traditional reviews and commentaries in that they cover studies by following an explicitly formulated procedure, which can help readers to understand the whole protocol followed for the literature review [227].

### Literature Search and Selection

The first step was identifying records. We conducted a systematic search of peer-reviewed literature (articles, reviews) using the key words in WOS and CNKI databases (Table 2). A total of 99 repeated references were excluded; 3821 references were selected for review. The search timeframe for both databases was the maximum timeframe of the databases, and the search deadline was 1 November 2022.

**Figure 5:**
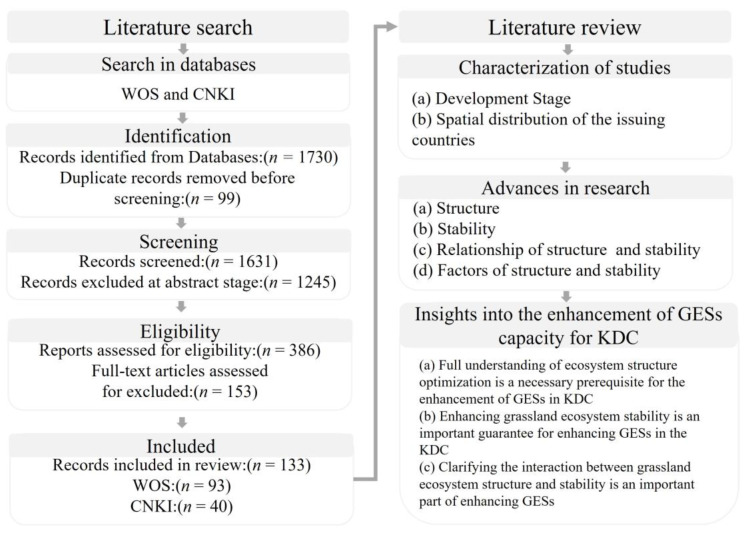
The flow diagram showing the steps of the methodology and selection process used for the systematic literature review on the left and the literature review process on the right.

The second step was screening. We screened the titles and abstracts of each article to select articles that assessed, depicted, and quantified or mapped grassland ecosystem structure and stability which were eligible for full-text reading (*n* = 278). The third step was reading the full text of each of the selected publications, and, finally, a total of 133 references were chosen as case studies. To summarize the information about main structural characteristics, stability, structure–stability relationship and influencing factors and suggest future directions of ecosystem service-capacity enhancement from case studies, we recorded the annual distribution of the literature, distribution of countries of publication, types of grassland structure, disturbance factors of grassland stability, and research methods (Figure 5).

## 4. Prospects

### 4.1. Key Scientific Issues That Need to Be Addressed

Research on the structure and stability of grassland ecosystems has achieved great success, but there are still many scientific questions that need to be addressed. Based on the previous systematic analysis, this paper categorizes the key scientific problems to be solved in the structure and stability of grassland ecosystem into three aspects: structure optimization and stability improvement and their relationship.

#### 4.1.1. Structure Optimization

To address the problems of ecological imbalance, food-chain disruption, and multiple functions in grassland ecosystems, we investigate the interactions between population dynamics and community properties through synergistic intra- and inter-species differences and spatial landscape-scale differences, determine the mechanisms and effects of biodiversity on the stability of ecosystem functions, and clarify the overall operation of ecosystems [27].

To address the problem of the relatively homogeneous grassland-ecosystem structure, this study suggests exploring the maintenance mechanisms of grassland-species allocation and stability; combining the heterogeneity of grassland functions and spatial patterns; performing a comprehensive assessment of grasslands in terms of local stand conditions, climatic habitats, plant functional traits, and other conditions; and targeting the selection of grass species with high adaptability and resistance to stress for mixed seeding [228].

To address the problem of the optimal configuration of grassland ecological structure, different grassland management measures can be combined to form an efficient and integrated management system by organically combining biological, engineering and management aspects to promote the sustainable development of grassland ecological animal husbandry. For example, scientific methods such as rotational grazing, rest grazing and limited-term enclosure, and the use of artificially planted forage instead of grazing. Sustainable grazing management in adaptive multiple paddocks (AMP) can be employed to incorporate forage and ruminants into regeneration-management planting systems to strengthen the productivity, stability and resilience of agroecosystems and, thus, improve ecosystem service capacity.

#### 4.1.2. Stability Improvement

The issue of the low stability of grassland ecosystems, can be explored through the mechanisms regulating the stability of grassland ecosystems, identify and quantify the factors affecting the stability of grassland ecosystems, select grass species suitable for local conditions, increase the diversity of grassland species, improve the resilience of grassland ecosystems, enhance ecological stability, and further promote the process of grassland ecological restoration, so as to reduce the ecological vulnerability of grassland and enhance the resilience and stability of grassland ecosystems [229].

To deal with the problem that the ecological stability index system and evaluation model have not been unified, it is necessary to establish and standardize the selection criteria of ecological stability evaluation indexes based on the analysis and summary of ecological stability research results, to select methods according to the basic attributes of grassland ecosystems, especially the key variables of ecosystem processes and functions, and to consider the validity, sensitivity and operability of alternative indexes. The evaluation indexes are selected using the expert consultation method, the evaluation index weights are determined using the hierarchical analysis method, and the mathematical model is constructed to specify them and quantify the factor percentages [230], so as to build a scientific grassland-ecosystem stability evaluation index system and promote the sustainable development of the system [231].

#### 4.1.3. Interaction between Structure and Stability

To address the problem of unclear relationships between the structure and stability of grassland ecosystems, study is based on the idea of “structure-process-function-services”, to strengthen the material cycle, energy flow and information transfer process of producer-consumer-decomposer in grassland ecosystems, to clarify the relationship between grassland species allocation and productivity and stability, and to clarify the response mode of structure and stability. It has been suggested that structure can directly alter the biodiversity of grassland ecosystems and further alter the stability of grassland ecosystems [232]. Therefore, the relationship between structure and stability can be explored through the medium of biodiversity.

To address the problem of single research methods (e.g., field surveys, field experiments) or models (statistics, modeling) for the structure and stability of grassland ecosystems, remote-sensing research into the process of interannual or monthly changes in grassland landscape patterns can be employed, combined with indoor experiments, field surveys, and field trials for comparative assessment [233,234].

### 4.2. The Current Status of Grassland Ecosystem for KDC and Inspiration for Improving the Ecosystem Service Capacity of Grasslands for KDC

#### 4.2.1. The Current Status of Grassland Ecosystem for KDC

Karst areas are very fragile and prone to ecological degradation due to their unique binary three-dimensional geological structure, of which karst desertification is an extreme manifestation of ecological degradation [229]. In the past 30 years, the KDC in southern China has achieved remarkable results in ecological environment and ecological restoration, but there is an urgent need to improve the stability of the KDC ecosystem and ecosystem service function [52,235]. Grasslands of the KDC are dominated by artificial grasslands and supplemented by improved grasslands, which constitute an important ecosystem in karst areas [229]. The artificial grasses in the KDC areas mainly include monoculture *Dactylis glomerata* L., *Lolium perenne* L. and *Medicago sativa* L. Grasslands have a monoculture planting structure and weak disease resistance, and the resulting artificial grass ecosystem is extremely fragile. This is mainly due to farmers’ pursuit of high forage yields. Thus, while the artificial grassland has brought high economic income to farmers for a short period of time, its ability to provide ecosystem services is gradually diminishing. In addition, the degraded grassland is dominated by replanting white clover by government aircrafts. However, due to the overgrazing behavior of farmers in recent years, the replanted degraded grassland is difficult to recover, and only some grass species that are resistant to gnawing and trampling are propagated, resulting in the difficult recovery of the grass species structure of degraded grassland and low stability of the ecosystem.

In the process of KDC and ecological restoration, grass is the pioneer plant for vegetation restoration and ecological environment improvement [64]. Therefore, optimizing the structure and enhancing the stability of grassland ecosystems for KDC is of great significance for the sustainable development of regional ecological environments and promoting the virtuous cycle of regional eco-industry.

#### 4.2.2. Inspiration for Improving the Ecosystem Service Capacity of Grasslands for the KDC

Due to its inherent sensitivity and fragility, the ecosystem structure and function of the grassland ecosystem in the KDC area are improper and the stability of the ecosystem is poor [236,237]. In the context of promoting the world’s desertification management and global sustainable greening, how to consolidate and stabilize the effectiveness of KDC and, at the same time, optimize the structure of desertification-control grassland ecosystems, guaranteeing the stability of the ecosystem and enhancing the ecosystem service capacity is a key issue that needs to be solved in the KDC area [238]. In view of this, based on the premise of sorting out the structure and stability of global grassland ecosystems, this paper drew on the above key scientific issues of structure optimization, stability enhancement and the interaction between structure and stability and, combined with the regional characteristics of karst desertification and the grassland status of KDC, proposes three insights for the enhancement of the ecosystem service capacity of grassland in the KDC area.

##### Adequate Understanding of Ecosystem Structure Optimization Is a Necessary Prerequisite for the Improvement of Ecosystem Services in Grasslands for the KDC

Although existing pasture cultivation has greatly increased forage production in the short term [239], it has made an outstanding contribution to KDC. However, the factors influencing the improvement of ecosystem services are not only determined by a single factor of production stability, and there is an urgent need to improve grassland ecosystem services through a variety of specific ways. Grassland ecosystem structure directly determines grassland ecosystem function and indirectly influences grassland ecosystem services. Research on multi-component mixes of legume grasses with different growth habits or morphological characteristics has concluded that the greater the number of components in the mix, the greater the likelihood of containing key species, and the greater the potential for community stability, thus achieving a seasonal balance in grassland output throughout the year and lasting stability in the number of years of use [240]. Similar experiments have been conducted in KDC areas, for example, the state replanted degraded grasslands with *Trifolium repens* L. and encouraged farmers to plant *Trifolium repens* L.+ *Lolium perenne* L. or *Dactylis glomerata* L. + *Medicago sativa* L. and other measures, which improved the ecosystem structure of grasslands of KDC to some extent. However, with the unique geographical environment of karst, seasonal drought and unreasonable human management, the ecological benefits of degraded and improved grasslands have gradually decreased [241]. Therefore, the following two points should be considered to enhance the service capacity of grassland ecosystems: (1) to optimize the planting structure of grassland ecosystems for rocky desertification management and enrich the spatial species composition of grassland, and to select stony, drought-tolerant and calcium-loving grasses for planting in response to the characteristics of karst desertification such as thin soil layers, easy soil erosion and calcareous lithology; and (2) strengthening research on the benefits of the structure–process–function–services cascade, so as to clarify the process of maintaining ecosystem productivity and stability [95].

##### Enhancing Grassland Ecosystem Stability Is an Important Guarantee for Enhancing Grassland Ecosystem Services in KDC

The more complex the structure of grassland ecosystem, the higher the degree of its ecological stability, which further affects the multifunctional functioning of grassland ecosystem and eventually changes the ecosystem service capacity. The existing grassland ecosystem structure of rocky desertification control is single and the grassland planting is mainly based on single high-yielding forage grasses, for example, *Pennisetum sinese Roxb* L. and *Lolium perenne* L., which have weak stability, low disease resistance and short yield supply time, which seriously hinders the sustainable development of local ecosystems and lacks effective measures to consolidate the achievement of rocky desertification control. Grassland ecosystem stability is an important factor that limits the sustainable development of grassland ecosystem productivity. Therefore, we should combine the needs of local farmers, and select suitable grass species for planting; conduct experiments on grass planting under different rocky desertification habitats; explore the best planting density of grass species and legume-grass planting ratio; and then improve the plant diversity of grassland ecosystems and enhance their stability and resilience. At the same time, we should combine various monitoring means (such as field sampling, drone aerial photography and remote sensing, etc.), and strengthen the research on the regulation mechanism of grassland ecosystem stability with the guidance of enhancing the sustainability of KDC, so as to improve the service capacity of the grassland ecosystems of KDC, further enhance its ecological recovery quality and maintain the sustainable development of the karst ecological environment.

##### Identifying the Interactions between Grassland Ecosystem Structure and Stability Is an Important Part of Enhancing Grassland Ecosystem Services

**Figure 6:**
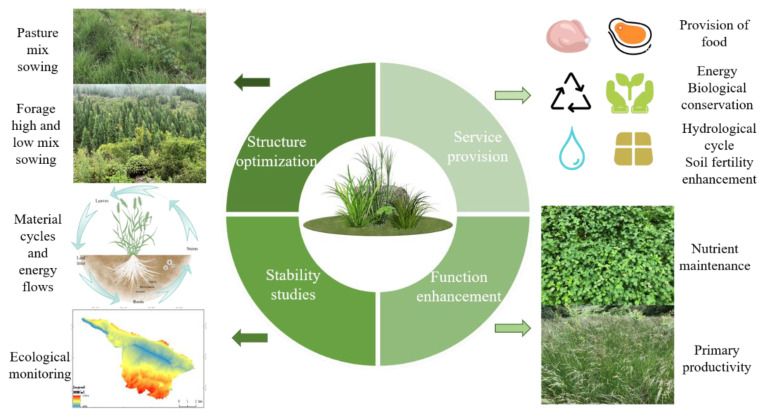
Processes of energy and service flows in grassland ecosystems (structure affects stability and thus changes the level of function and ultimately service provision).

This paper showed that most of the literature focuses on multiple factors of the structure and stability of grassland ecosystems, but these studies have mainly focused on unidirectional effects, such as Gschwend et al. and Hu et al. showing that biodiversity can stabilize productivity through different mechanisms, such as the asynchronous responses of species to environmental change and stability [242,243]. What is more, exploring the mutual response mechanisms between grassland planting structures and their productive and ecological stability is an important element in clarifying the effects of current grass seed mixes in KDC and local sustainable development. Xu et al. explored the water–fertilizer coupling mechanism between soil and forage through intercropping in the southern karst region. The experiments proved that a reasonable intercropping system could coordinate the relationship between crops and environment and improve soil nutrients, which made a certain contribution to exploring soil–plant relationships in the grasslands of karst areas and improving the structure and stability of stone desertification grassland ecosystems [244]. However, from the perspective of the interaction mechanism of grassland ecosystem structure and stability, exploration into the changes in the supply of ecosystem service capacity of grassland managed by karst rock desertification is still lacking. Therefore, through field control experiments in the context of KDC, the interaction mechanisms of grassland-ecosystem structure and stability should be clarified in the context of KDC, and the exploration of above-ground-subsurface material flow and nutrient circulation fluency should provide important insights to elucidate the trade offs and synergistic relationships among service flows (Figure 6).

## 5. Conclusions

Based on the WOS and CNKI databases, this study systematically reviewed the research progress of grassland-ecosystem structure and stability, and concluded the following: (1) There is a significant increase in the number of annual publications on the structure and stability of grassland ecosystems, and the research directions and themes are becoming increasingly diverse. The research mainly focuses on revealing the mechanisms influencing species diversity and stability, quantitatively studying the nutrient and productivity of forage grasses, and the regulatory mechanisms of stability. (2) The spatial pattern of the study countries is highly consistent with the spatial distribution characteristics of grassland ecosystems, mainly dominated by countries with wider grassland areas such as China and the United States. However, the number of national publications in Africa contradicts the distribution pattern of grasslands. (3) The research on the structure and stability of grassland ecosystems focuses on the structural characteristics of grassland ecosystems, structural optimization, ecosystem stability, and the structure–stability relationship and its influencing factors, in which the grassland-ecosystem structure and structure and stability influencing factors are the main research directions (4) The key scientific issues that need to be addressed nowadays were grouped according to whether they addressed structure optimization, stability enhancement, and structure–stability relationship. We should strengthen the multi-perspective exploration of grasslands under different spatial and temporal conditions, to enhance the functional output of grassland ecosystems from optimizing the structure of grassland ecosystems, to enhance the stability of grassland ecosystems, and to form a structure–process–function–services cascade benefit study. Based on the above conclusions, three insights can be provided for the enhancement of grassland ecosystem services in the KDC: (i) fully understanding ecosystem structure optimization is a necessary prerequisite for karst grassland stability; (ii) enhancing grassland ecosystem stability is an important guarantee for enhancing grassland ecosystem services in KDC; and (iii) clarifying the interaction between grassland ecosystem structure and stability is an important link to enhance grassland ecosystem services.

## Figures and Tables

**Table 1:** Distribution of the number of publications issued by countries or region.

Country or Region	Number of Articles Issued
China	53
American	22
Australia	8
Canada, England	6
Germany, Switzerland	5
France, The Netherlands	4
South Africa, Brazil	3
Estonia, Poland, Austria, Japan, Mexico, Sweden, Senegal, New Zealand, Belgium, Argentina, Denmark, Romania, Spain, Italy	1

**Table 2:** Literature search terms and results.

Literature Databases	Types	Search Terms (All Fields)	Number of Initially Acquired Publications
WOS and CNKI	Structure	“Grassland” OR “Meadow” OR “Pasture” OR “Rangeland” OR “Steppe” OR “Prairie” OR “Veld” OR “Savanna” AND “Ecosystem structure” “Food chains” OR “Food web” OR “Trophic levels” OR “Nutrient levels” OR “Community structure” OR “Species configuration” OR “Biodiversity” OR “Landscape pattern” OR “Forage mixes” OR “ecological corridors” OR “Time change” OR “Interannual variation” OR “Month change” AND “Ecosystem services”	1435
	Stability	“Grassland” OR “Meadow” OR “Pasture” OR “Rangeland” OR “Steppe” OR “Prairie” OR “Veld” OR “Savanna” AND “Ecosystem stability” OR “Stability assessment” OR “Flexibility” OR “Resistance” OR “Resilience” AND “Ecosystem services”	295
Total			1730

## Data Availability

Not applicable.
